# Flash Suppression Reveals an Additional Nonvisual Extrastriate Contribution for Amblyopic Suppression

**DOI:** 10.1167/iovs.65.2.41

**Published:** 2024-02-28

**Authors:** Dave Saint-Amour, Laura Lefebvre, Clémence Bertrand Pilon, Robert F. Hess

**Affiliations:** 1 Department of Psychology, Université du Québec à Montréal, Montreal, Quebec, Canada; 2 Department of Ophthalmology, Université de Montréal, Montreal, Quebec, Canada; 3 Research Center, CHU Sainte-Justine, Montreal, Quebec, Canada; 4 Clinique de Neuropsychologie, Centre Hospitalier Universitaire de Sherbrooke, Sherbrooke, Quebec, Canada; 5 Department of Psychology, Université de Montréal, Montreal, Quebec, Canada; 6 Department of Ophthalmology and Visual Sciences, McGill University, Montreal, Quebec, Canada

**Keywords:** amblyopia, suppression, visual evoked potential, visual psychophysics

## Abstract

**Purpose:**

A growing body of evidence suggests that anomalous binocular interactions underlie the deficits in amblyopia, but their nature and neural basis are still not fully understood.

**Methods:**

We examined the behavioral and neural correlates of interocular suppression in 13 adult amblyopes and 13 matched controls using a flash suppression paradigm while recording steady-state visual evoked potentials. The strength of suppression was manipulated by changing the contrast (10%, 20%, 30%, or 100%) of the flash stimulus, or the suppressor, presented either in the dominant (fellow) or nondominant (amblyopic) eye.

**Results:**

At the behavioral level, interocular suppression in normal observers was found, regardless of the eye origin of the flash onset. However, the pattern of suppression in the amblyopes was not symmetric, meaning that the suppression from the dominant eye was stronger, supporting a putative chronic suppression of the amblyopic eye. Interestingly, the amblyopic eye was able to suppress the dominant eye but only at the highest contrast level. At the electrophysiology level, suppression of the steady-state visual evoked potential responses in both groups in all conditions was similar over the occipital region, but differed over the frontal region.

**Conclusions:**

Our findings suggest that, although suppression in amblyopia involves an imbalanced interaction between the inputs to the two eyes in the visual cortex, there is also involvement of nonvisual extrastriate areas.

Amblyopia is a neurodevelopmental disorder resulting from the disruption of the binocular visual experience during early childhood that has an incidence of approximately 3%. Even though amblyopia is typically diagnosed from interocular difference of visual acuity, there is also reduced contrast sensitivity to high spatial frequencies,^1^^–^^3^ as well as deficits in orientation,^4^ global motion processing,^5^^,^^6^ and stereo vision.^7^ Furthermore, impairments of high-order functions have been reported in several domains, including visual attention,^8^^–^^12^ prehension movements,^13^ face recognition,^14^^–^^16^ reading,^17^^,^^18^ and decision-making.^19^

Of the many deficits that are associated with amblyopia, there is reason to believe that the loss of binocularity is the most important from an etiological perspective.^20^ Although it was once believed that the primary problem was a loss of monocular function with the loss of binocularity being a consequence,^21^ there is now evidence that the primary problem is a loss of binocularity with poor monocular function being the consequence of chronic suppression.^22^ Also, it has been suggested that the structural integrity of binocular vision remains intact in amblyopia, but it is rendered functionally monocular due to suppression of the amblyopic eye by the fellow sighted eye.^23^ Therefore, a better understanding of these suppressive influences is paramount if we are to understand the neural basis of amblyopia and know best to treat it.

Initially, it was thought that the normal fellow fixing eye exerted abnormally large inhibition of the amblyopic eye^21^; however, as the reciprocal nature of the binocular circuit before combination became better understood,^24^ it was recognized that a net imbalance in these reciprocal interactions may underlie suppression in amblyopia. It was shown that there was a normal inhibitory response emanating from the normal fixing fellow eye, but a much reduced inhibitory response emanating from the amblyopic eye, with the net interaction favoring the fellow sighted eye.^25^ One early proposal to explain why the inhibitory response from the amblyopic eye is weaker was to suggest it was the result of the reduced monocular input,^26^ the so-called attenuator hypothesis. However, it was later shown that suppression, as revealed by a static spatial masking paradigm, is greatest at low spatial frequencies where the monocular attenuation (i.e., contrast deficit) is at its weakest.^25^^,^^27^ It is presently assumed that that suppression involves an inhibitory imbalance at the level of the thalamocortical synapse^28^ or at the level of the reciprocal cortical binocular interactions themselves.^29^

The site of suppression has traditionally been thought to be in the striate cortex, because this is where integration of the monocular information occurs. There is evidence from neurophysiological studies in cat cortex that, at least at the single cell level, strong inhibitory signals affecting the amblyopic eye input have been documented^29^^–^^31^ and that these may be located in layer 4 of V1^28^ or in V2.^32^ In humans, there is functional magnetic resonance imaging evidence both for^33^ and against^34^ a striate site of suppression. There is also a suggestion that, although the effects are seen in V1, the origin may be in extrastriate cortex and involve a feedback deficit of attentional modulation of the amblyopic eye.^35^ The site of such selective attentional modulation is thought to be in posterior parietal cortex^36^ or prefrontal cortex.^37^ A critical issue at present is whether suppression in amblyopia is limited to the early visual areas (striate cortex or extrastriate cortex, V1, V2 ) or whether it also involves feedback deficits to higher, nonvisual cortical areas (such as posterior parietal or prefrontal cortex).

Here we use a temporal masking approach to address the nature and site of suppression. Much of what we know of suppression comes from the noise-masking approach using static spatial stimuli,^25^ so we wanted to know if this could be generalized to the temporal domain. Temporal masking involves transient stimulation and by definition involves low spatial frequencies and mid to high temporal frequencies (8 Hz) for which there is no significant monocular deficit,^38^ thus providing a strong test of the attenuator hypothesis of masking.^26^ Also, there are documented differences between spatial and temporal masking in amblyopia when it comes to orientational selectivity,^39^^,^^40^ so there is every reason to expect different results on suppression for these two types of masking. The second reason for undertaking this study was to assess whether suppression in amblyopia is limited to striate^28^^,^^31^ or early extrastriate areas^32^ or whether higher non visual cortical areas are also involved.^35^ We used a steady-state visual evoked potential (ssVEP) mapping approach with our behaviorally validated flash masking approach to investigate the neural correlates of the suppressive interactions in amblyopia.

## Methods

### Participants

All participants gave informed consent, in accordance with the CHU Sainte-Justine ethics board. Thirteen observers with normal or corrected vision (mean age = 27. 9 years, 6 males) and 13 amblyopic observers (mean age = 31.1 years, 4 males) participated in the experiments. Of the amblyopic participants 11 were strabismic, 1 anisometropic, and 1 microstrabismic with anisometropia. The presence of strabismus was confirmed with the cover test, a common clinical technique to assess eye misalignment. Anisometropia was defined as interocular difference in spherical equivalent refraction of 1.5 diopters or more. All participants were required to wear their best optical correction (if any). An interocular difference of 0.2 logMAR, which is generally considered clinically significant for amblyopia, was present in all amblyopic participants. Ocular dominance was measured with the Porta test, which determines which monocular view of a near target best matches the binocular view. Corrected visual acuity was measured monocularly with a logMAR chart presented at 3.05 m. Stereopsis threshold was assessed with the Stereo Fly Test (Stereo Optical Co., Chicago, IL, USA). Clinical details of the amblyopes are provided in Table.

**Table.** Clinical Details of Amblyopic Participants.

ID	Sex	Age, Years	Condition	VA DE	VA NDE	Stereo, Arcsec
01	F	28	Strabismus	0	0.2	3000
02	F	26	Microstrabismus + anisometropia	0.1	0.3	40
03	F	29	Strabismus	−0.1	0.4	400
04	F	30	Strabismus	−0.1	0.5	50
05	F	20	Strabismus	0.2	0.4	100
06	M	49	Strabismus	0	0.4	200
07	F	54	Strabismus	0.1	0.4	>3000
08	F	34	Anisometropia	0	0.5	80
09	F	27	Strabismus	−0.1	0.4	>3000
10	M	29	Strabismus	−0.1	0.4	400
11	F	30	Strabismus	0.1	0.6	>3000
12	M	29	Strabismus	0	0	200

DE, dominant eye; NDE, nondominant eye; VA, visual acuity.

### Stimuli and Procedure

Two sinusoidal gratings (10° of visual field) with a spatial frequency of 1 cycle per degree (cpd) were presented dichoptically through a virtual reality display (Model Z800 3DVisor; eMagin Corp. Bellevue, WA, USA), driven by a MAC G4 Desktop with an NVIDIA graphics card (Geforce 9400m, Santa Clara, CA, USA). Each monocular resolution organic light-emitting diode screen had a resolution of 800 × 600 pixels. The refresh rate was 60 Hz and the visual field was 32° × 23° for each eye. The device allowed for fitting over existing correction devices (or eyewear) and adjusting the center-to-center distance between left and right eye screens to match the interpupillary distance of each subject. Screen pixel size subtended 2.4 min of arc at the eye and the mean luminance was 60 cd/m^2^.

**Figure 1.**
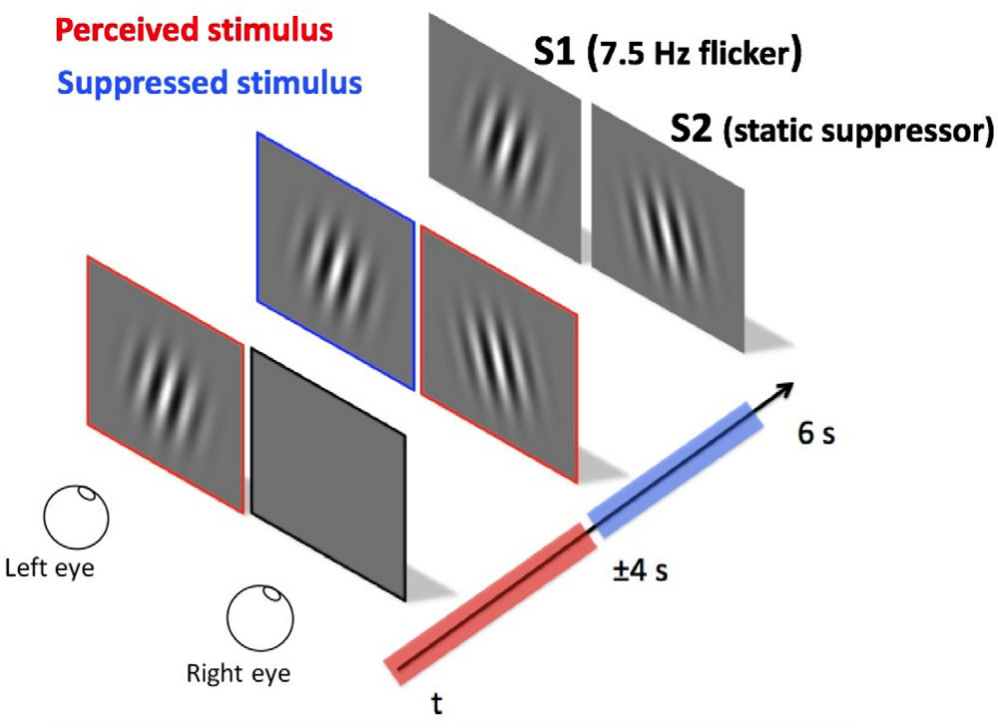
Flash suppression dichoptic presentation over time. S1 = sinusoidal grating of 1 cpd, 30% contrast flickered at 7.5 Hz. S2 = sinusoidal grating of 1 cpd, 10%, 20%, 30%, or 100% contrast and static (0 Hz).

The first grating (1 cpd) stimulus (S1) flickered at a temporal frequency of 7.5 Hz (15 reversals/second) with a contrast of 30%, kept constant. S1 was presented to one eye with an 85° or 95° orientation for 3.5 to 4.5 seconds (jitter duration with a mean of 4 seconds) while the other eye viewed a grey screen. The second grating (1 cpd) stimulus (S2) was presented to the other eye for 2 seconds. S2 was static (no flicker) with the orientation reversed (i.e., one eye at 85° and the other at 95°, or vice versa). The total duration of each trial was 6 seconds (4 seconds monoptic and 2 seconds dichoptic). The orientation (85° or 95°) was counterbalanced between the trials and stimuli were presented at four contrast levels (10%, 20%, 30%, and 100%). Each condition was repeated 15 times for each eye, for a total of 120 trials. During the task, participants were instructed to report the stimulus orientation (85°, 95°, or mixed orientation) by pressing one of the three corresponding buttons. Partial (mixed orientation) and total (85° or 95° orientation) percepts were grouped together as a measure of suppression. The procedure is illustrated in Figure 1.

### Suppression Measurements

Interocular suppression was estimated using two metrics, which served as dependent variables. First, we calculated the time it took from the S2 flash onset to a perceptual change, that is, the suppression of S1, as indicated by a button press. In complement with this latency suppression metric, a second metric was derived by computing the duration of the suppression. For instance, if the suppression appeared 0.5 seconds after the S2 and lasted 1.5 seconds, a value of 0.5 was assigned to the latency metric and 1.5 to the duration metric. If no suppression at all was detected for a given trial, a value of 2 (end of the trial) was assigned to the latency measure and 0 to the duration measure. The duration measure was expressed as the ratio between the perceived suppression duration and the S2 presentation duration (2 seconds). Therefore, a ratio of 1 was the maximum amount of suppression.

ssVEPs were recorded simultaneously with psychophysical measures using a 64-channel active technology EEG system (QuickAmp, Brain Products GmbH, Munich, Germany). Discrete Fourier transformation was applied to the electrophysiological data to extract the amplitude of the 7.5-Hz flickering stimulus (S1) at the second harmonic (i.e., 15 Hz). The discrete Fourier transformation amplitude was used to compute an interocular suppression index, according to the following formula: [(Monoptic response – Dichoptic response)/Monoptic response]. Trials under monoptic stimulations with 3 or more SD of the mean and/or signal-to-noise ratio of 2 or less were excluded from the analysis.

### Data Analysis

Statistical analyses were carried out using SPSS software package version 22.0 (SPSS Inc., Chicago, IL, USA). In all analyses of variance (mixed-design repeated-measures ANOVAs), if the sphericity assumption was violated, Greenhouse–Geisser corrections were reported. Follow-up ANOVAs were conducted to decompose interaction effects. In all ANOVA models, Bonferroni correction was applied to control for multiple comparisons. Spearman correlations (bilateral) were used to test the associations between the behavioral and ssVEP suppression indexes.

An electroencephalography (EEG) signal was collected with a band pass of 0.1 to 100.0 Hz at a rate of 500 Hz. Trials with blinks and eye movements were corrected using an independent component analysis approach,^41^ which removes eye-related artefacts from the EEG without excluding any trials. An amplitude criterion of ±200 µV was used to reject trials with other artefacts (head movement, muscular contradiction, etc.). EEG analyses were performed with *Analyzer 2* software (Brain Products, Inc., Munich, Germany).

Two regions of interest (ROI) were defined based on the topographic voltage distribution from the nonamblyopic group (both eyes averaged) obtained at maximal stimulus contrast level (see Fig. 4A). The occipital ROI (the average of Oz, O1, O2, and POz) included the electrodes surrounding the midline electrode having the maximal amplitude, namely, Oz. The frontal ROI (average of Cz, FCz, Fz, FC1, and FC2) included the electrodes surrounding the midline electrode showing the maximal response, namely, FCz.

## Results

### Visual Acuity

**Figure 2.**
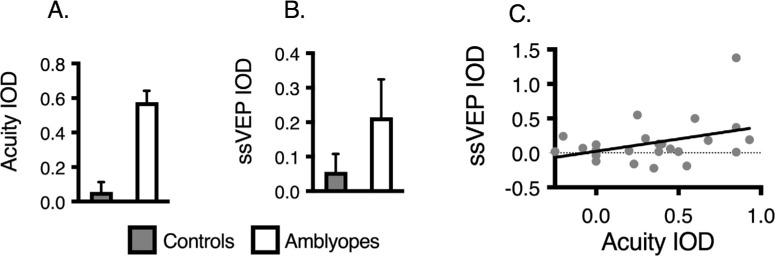
(**A**) Acuity interocular difference in controls vs. amblyopes. (**B**) Occipital ssVEP interocular difference in controls vs. amblyopes. (**C**) Correlation between acuity interocular difference and ssVEP interocular difference. IOD = interocular difference. Error bars = standard error of the mean.

As illustrated in Figure 2, the interocular difference of subjective acuity between groups was significantly larger in the amblyopes than in the control groups, F (1, 24) = 35,91, *P* < 0.0001, η^2^ = 0.60. Although the interocular difference of ssVEP responses at Oz was also larger in the amblyopes, no significant difference was found in comparison with the controls, F (1,24) = 1.68, *P* = 0.21, η^2^ = 0.07. This finding is likely due to our stimulus spatial parameters (low spatial frequency and large visual field), which were chosen to evoke good responses even in the amblyopes. Although a positive correlation was observed between acuity and ssVEP amplitude (r = 0.39, *P* = 0.048), it was modest, suggesting that subjective acuity is not explained fully by occipital cortex activity.

### Behavioral Interocular Suppression

**Figure 3.**
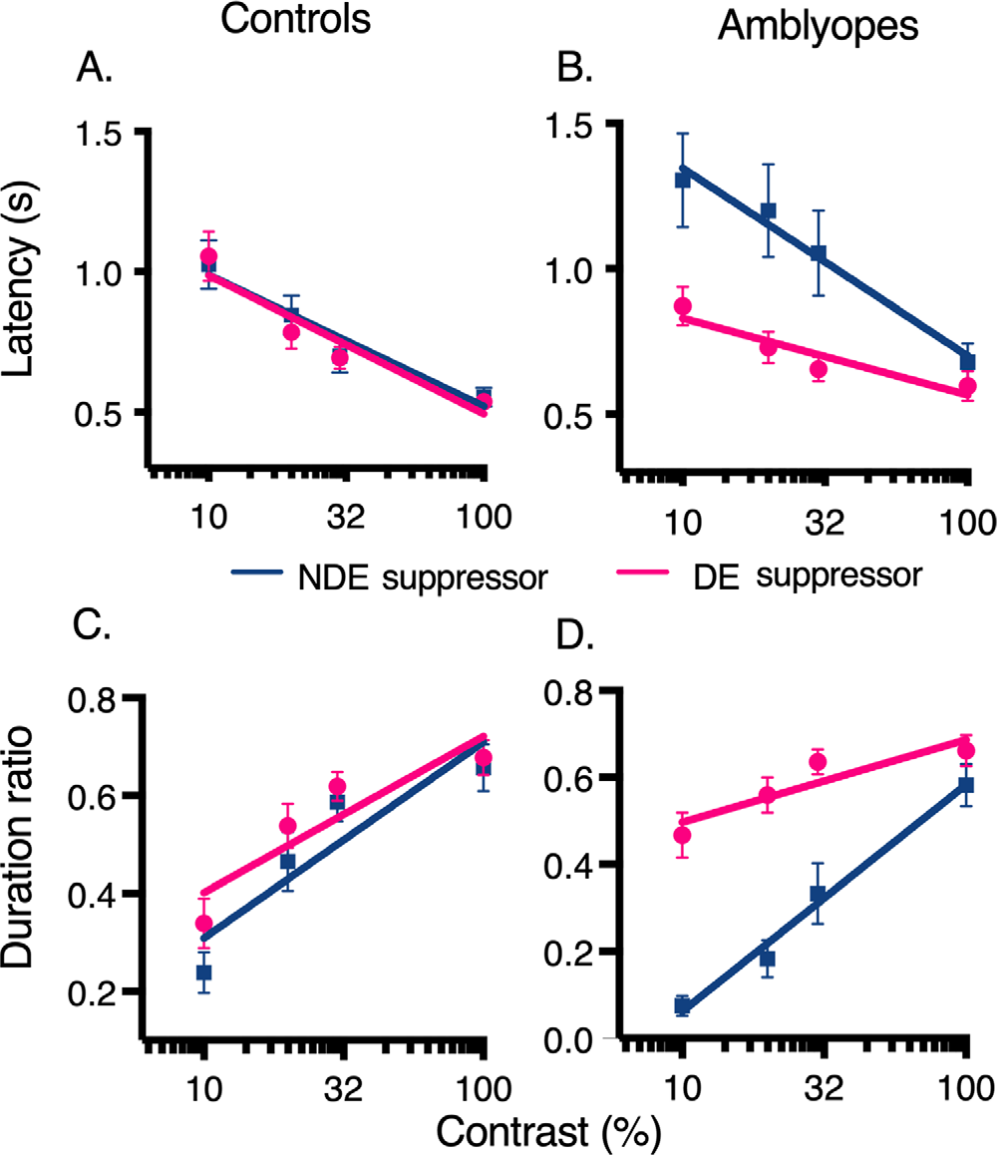
Onset latency and duration ratio suppression in controls (**A**, **C**) and amblyopes (**B**, **D**). Responses are shown when the flash suppressor come from the dominant/fellow eye (DE) or the nondominant/amblyopic eye (NDE). Error bars = standard error of the mean.

Separated mixed design repeated-measures ANOVAs were conducted, with eye (dominant or nondominant), contrast (10%, 20%, 30%, or 100%) and group (amblyopic or control) as factors for each suppression measure (onset latency or duration ratio) A significant three-way interaction effect between eye, contrast and group, F (2.62, 62.79) = 2.97, *P* = 0.045, partial η^2^ = 0.11, was found for onset latency (Figs. 3A, 3B). Follow-up ANOVAs in control observers showed a main effect of contrast, regardless of which eye, that is, the dominant or nondominant eye, F (1.28, 15.39) = 36.44, *P* = 0.000008, partial η^2^ = 0.75, so that latency decreased linearly as a function of contrast. In amblyopic observers, results revealed a significant interaction between eye and contrast, F (2.62, 31.44) = 3.478, *P* = 0.032, partial η^2^ = 0.23 (see Fig. 3B), so that suppression latency was longer when the amblyopic eye suppressed the dominant eye at 10%, 20%, and 30% contrast (*P* < .05), but was similar between eyes at 100% contrast (*P* > 0.05), so that interocular suppression was similar, regardless of the origin of the flash suppressor. In other words, the dominant eye was generally only weakly suppressed by the amblyopic eye, but not at the 100% contrast condition, at which level the amblyopic eye was effective in suppressing the dominant eye.

**Figure 4.**
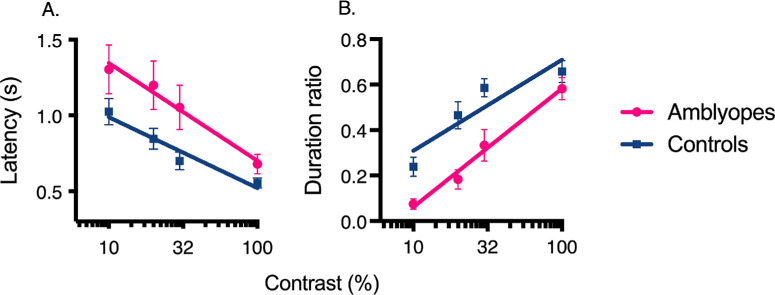
Comparison of the responses of the fellow fixing eye of amblyopes and the dominant eye of controls for latency measure (**A**) and duration ratio measure (**B**). Error bars = standard error of the mean.

Regarding duration (Figs. 3C and 3D), results showed a significant three-way interaction effect between eye, contrast and group, F (2.55, 61.13) = 4.40, *P* = 0.01, partial η^2^ = 0.155 (see Fig. 4). In controls, follow-up ANOVAs revealed a main effect of contrast, F (3, 36) = 37.88, *P* < 0.0001, partial η^2^ = 0.759 (Fig. 3C), regardless of eye origin. Suppression duration increased linearly as a function of contrast. In amblyopes, a significant interaction between eye and contrast was found, F (2.36, 28.37) = 10.59, *P* < 0.0002, partial η^2^ = 0.47 (Fig 3D). Suppression duration was significantly higher when the dominant eye suppressed the amblyopic eye at 10%, 20%, and 30% contrast, suggesting chronic suppression. This asymmetry was not observed at the 100% contrast level, both eyes having an equivalent suppression capacity at this level.

Another mixed ANOVA with a within-subject factor of contrast and a between-subjects factor of eye (fellow fixing eye vs. control eye) were conducted for each suppression measure to compare responses of the fellow fixing eye of amblyopes with the dominant eye of controls (Fig. 4). Regarding onset latency, results showed a main effect of contrast, F(3, 69) = 24.73, *P* < 0.001, partial η^2^ = 0.507, meaning that latency decreased linearly as a function of contrast, regardless of groups. Similar for duration, a main effect of contrast was found, F(3, 69) = 72.14, *P* < 0.001, partial η^2^ = 0.75, indicating that duration also increased linearly as a function of contrast. Compared with controls, suppression was decreased in the fellow fixing eye of amblyopes for both measures and this effect was statistically significant at all contrast levels, except 100%.

### Electrophysiological Interocular Suppression

**Figure 5.**
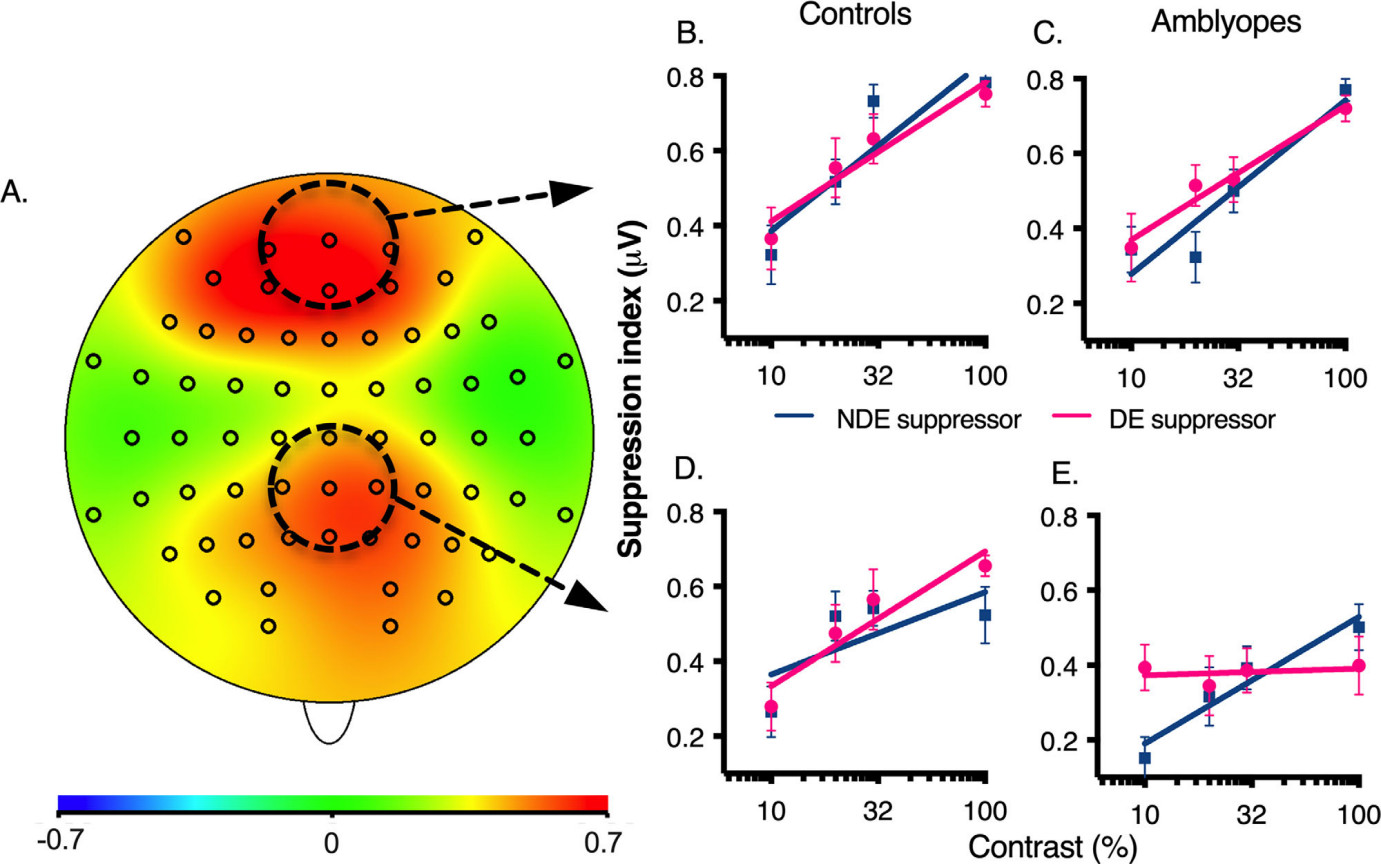
(**A**) Topographic distribution of cortical suppression in controls. (**B**) Suppression in occipital ROI of controls. (**C**) Suppression in occipital ROI of amblyopes. (**D**) Suppression in frontal ROI of controls. (**E**) Suppression in frontal ROI of amblyopes. DE = dominant eye; NDE = nondominant eye. Error bars = standard error of the mean.

Neural correlates to the results above were explored using high-density EEG. A mixed-design (ROI × Eye × Contrast × Group) repeated-measures ANOVAs was conducted on the ssVEP suppression index. Results revealed a significant four-way interaction, F (3, 72) = 3.05, *P* = 0.034, partial η^2^ = 0.113 (Fig. 5). For the occipital ROI, a follow-up ANOVA indicated a main effect of contrast, F (1.84, 44.18) = 42.959, *P* < 0.0001, partial η^2^ = 0.642, meaning that suppression increased linearly as a function of contrast (see Figs. 5B and 5C). Surprisingly, this effect was similar for both eyes and groups.

Regarding the frontal ROI, follow-up ANOVAs showed a three-way interaction between group, eye, and contrast, F (3, 72) 2.743, *P* = 0.049, partial η^2^ = 0.103 (see Fig. 5). In controls, a main effect of contrast was found, F (3, 36) = 15.18, *P* < 0.0001, partial η^2^ = 0.559 (see Fig. 5D). Suppression increased linearly as a function of contrast. As expected, no eye dominance effect was found. In amblyopes, ANOVAs revealed a significant interaction between eye and contrast, F (3, 36) = 4.850, *P* = 0.006, partial η^2^ = 0.288 (see Fig. 5E), where suppression in the amblyopic eye was evident even at the lowest contrast level (see Fig. 5E).

### Correlations Between Behavioral and Electrophysiological Measures

**Figure 6.**
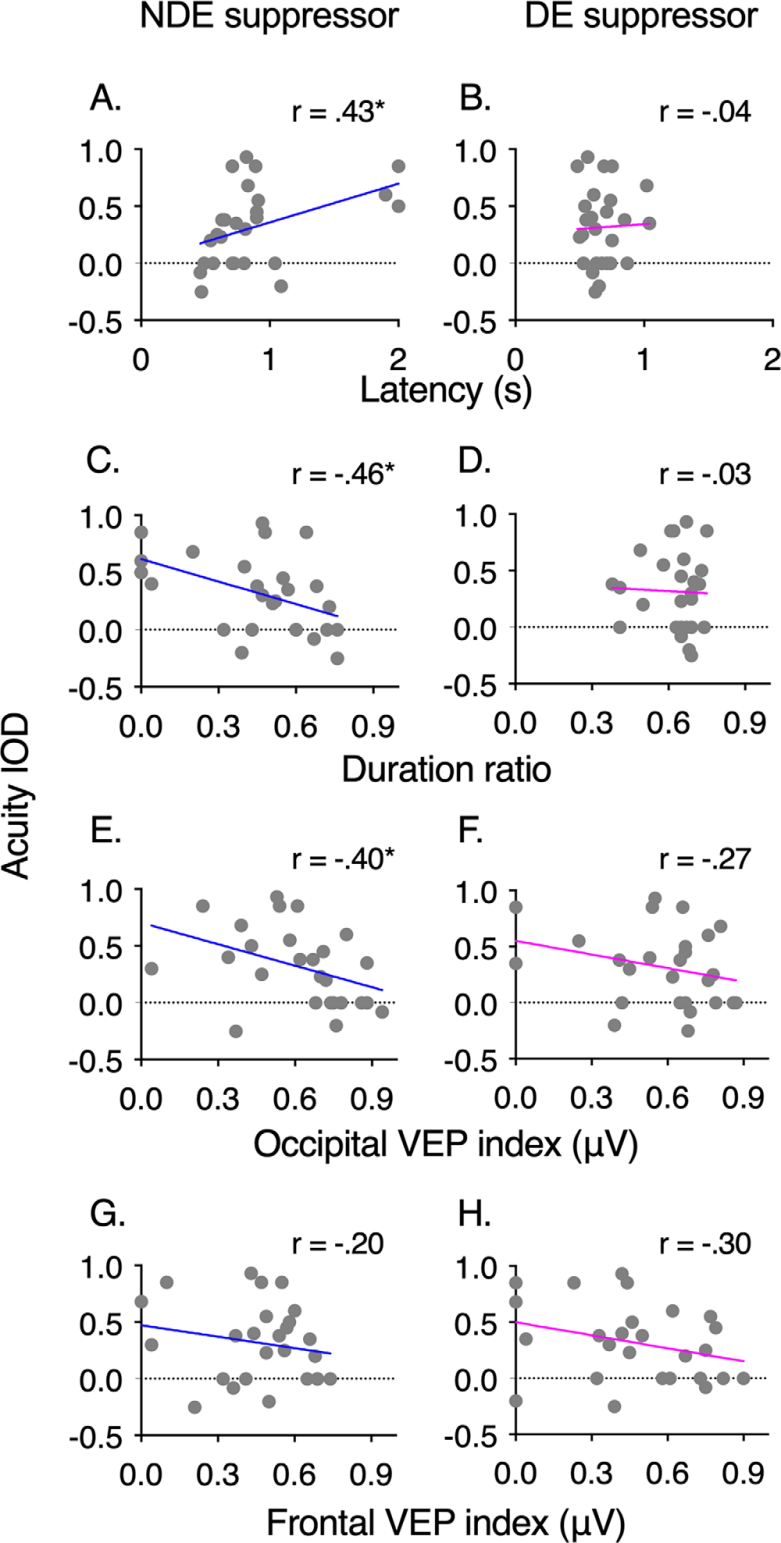
Correlation between acuity interocular difference and suppression from nondominant/dominant eye at 30% of contrast according to (**A**, **B**) reaction time, (**C**, **D**), duration ratio, (**E**, **F**) occipital suppression, and (**G**, **H**) frontal suppression. IOD = interocular difference; DE = dominant eye; NDE = nondominant eye. Error bars = standard error of the mean.

Correlation analyses between behavioral and electrophysiological measures at equivalent contrast condition, that is, when both eyes were stimulated at the same contrast level (30%), were conducted in all participants. The interocular difference of acuity between the eyes was correlated with the perceptual (Figs. 6A–6D) and ssVEP (Figs. 6E–6H) suppression indexes. When the dominant eye was suppressed by the nondominant eye, interocular acuity difference was positively correlated with suppression latency (r = 0.43, *P* < 0.05), that Is, the more acuity difference increased, the more latency increased (Fig. 6A). In agreement with this result, acuity difference was negatively correlated with suppression duration (r = −0.46, *P* < 0.05), that is, the more the acuity difference increased, the more duration ratio decreased (Fig. 6C). Correlations with the ssVEP suppression indexes showed that acuity difference was correlated negatively with the occipital ROI (r = −0.4, *P* < 0.05), but not with the frontal ROI suppression (r = −0.2, *P* = 0.34). These correlations indicated that the more the nondominant eye suppresses the dominant eye, the more the acuity is equivalent between eyes. When the nondominant eye was suppressed by the dominant eye (Figs. 6B, 6D, 6F, and 6H), neither of the behavioral and electrophysiological measures were correlated with acuity difference (latency: r = 0.04, *P* = 0.84; suppression duration: r = −0.03, *P* = 0.90; occipital ROI: r = −0.27, *P* = 0.18; frontal ROI: r = −0.3, *P* = 0.14).

## Discussion

The aim of this study was to investigate the nature and the site of interocular suppression in amblyopia using the flash suppression paradigm with two temporally based metrics (suppression onset latency and duration). These measures were obtained using a low spatial frequency flash, providing a new and complimentary assessment of suppression to that already published using static noise masking^25^ or temporally varying spatial noise masks.^39^ At the behavioral level, we found that interocular suppression in control observers was not dependent of the eye origin of the flash stimulus. In amblyopes, however, suppression was asymmetric in such a way that suppression was stronger from the dominant eye, suggesting chronic net suppression of the amblyopic eye. Interestingly, the amblyopic eye was able to suppress the dominant eye when stimulated with a relatively higher contrast, suggesting that the amblyopic eye at lower contrasts produced a weaker suppression of the sighted eye than vice versa.^23^ A comparison of the suppression in the fixing eyes of amblyopes versus the dominant eyes of controls also confirmed that the fixing eye does not initiate abnormally high levels of suppression relative to a normal eye; if anything, the sighted eye of amblyopes has lower levels of suppression compared with normal eyes. This result using transient stimulation and a temporal masking metric is in line with previous psychophysical studies involving static spatial masking and a contrast metric.^25^ First, the basis of the amblyopic eye being suppressed stems from a poorer inhibition of the sighted eye by the amblyopic eye rather than vice versa.^25^ Second, binocular combination can occur when the contrast presented to the sighted eye is decreased,^42^ because this factor redresses the net inhibitory imbalance between the sighted and amblyopic eyes. The fact that these two quite different psychophysical approaches each with their distinctly different metrics agree on these key issues provides support for our current understanding of the basis of suppression being caused by an imbalance in the reciprocal inhibitory interactions that underlie binocular combination as a result of a weaker suppression of the sighted eye by the amblyopic eye.

At the electrophysiological level, suppression of the ssVEP responses in both groups, regardless of the eye, was very similar over the occipital ROI, but differed for the frontal ROI. The former was unexpected because there is neurophysiological evidence in strabismic animals for a striate site of suppression.^28^^,^^43^ The latter was unexpected because it suggests that nonvisual higher level brain areas play a role in interocular suppression in amblyopia. Also, by contrast with the controls, frontal ssVEP suppression of amblyopic eye did not depend on the contrast of the suppressing stimulus.

### Interocular Differences

By contrast with the subjective acuity, the difference of interocular monoptic ssVEP responses at Oz was not statistically different between the amblyopic and control observers (Fig. 2). Although electrophysiological interocular differences between amblyopic and fellow eyes has been reported in the literature commonly,^44^^–^^48^ some authors have also shown no electrophysiological difference between the amblyopic and fellow eyes.^49^^–^^51^ Stimulus parameters are likely to be the major factor responsible for these discrepant findings. In support of this finding, it has been reported that a pattern checkerboard VEP is more efficient than an unpatterned stimulus for revealing interocular differences in amblyopia.^45^ Furthermore, low temporal frequency and high spatial frequency are more likely to reveal interocular differences.^44^^,^^46^ Thus, the fact that we used a relatively low spatial frequency stimulus (1 cpd) is most likely the reason the monopic ssVEP were found to be similar between normal and amblyopic eyes.

As expected, both groups differed in terms of interocular subjective acuity, which often reflects the severity of suppression.^22^ We showed that interocular difference of acuity was correlated with the behavioral evidence of suppression (onset latency and duration ratio) when the nondominant/amblyopic eye was suppressing the dominant eye (Figs. 6A and 6C), but not the reverse, that is, when the nondominant/amblyopic eye was suppressed by the dominant eye. In the latter case (Figs. 6B and 6D), the magnitude of suppression was higher, which may have decreased data variability, thus preventing regression modeling. The results must nevertheless be interpreted with caution, because the variability in the data and the low number of participants in our study might have decreased the goodness of fit of our correlation models.

### Neural Correlates of Amblyopic Suppression

Amblyopia is commonly associated with an interocular chronic suppression.^52^ Several studies have suggested that V1 is the locus of suppression in amblyopia because suppressive interactions have been demonstrated in binocular neurons in V1,^31^ and it has been argued that inhibitory interactions occur between thalamic inputs and V1 cells.^28^ There is also evidence in primates that interocular suppression is more evident in the cellular responses in V2 rather than V1.^32^ In humans, where the assessment is more indirect, there is evidence both for^33^ and against a V1/V2 locus for suppression using functional magnetic resonance imaging.^34^ Finally, there is a suggestion that a lack of attentive control specific to the amblyopic eye from higher visual areas may be responsible for any apparent deficit in V1/V2.^35^

In the present study, perceptual interocular suppression in amblyopes clearly differed from controls because the dominant (fellow) eye was more effective in suppressing the nondominant (amblyopic) eye, whereas both eyes behaved similarly in controls. In contrast with this expected result reflecting chronic suppression, we did not find evidence of abnormal suppressive processing in the occipital region of amblyopes; the ssVEP occipital responses in amblyopes were similar to those of the controls. This finding does not rule out that the binocular architecture in the early visual cortex may be responsible for amblyopic suppression, because any asymmetry differences in the activation of reciprocal inhibitory networks, as has been suggested in amblyopia, may be beyond the resolution of our ssVEP approach. Although it is not controversial that the amblyopic deficit may not be confined to area V1,^53^^,^^54^ it is more controversial that the locus of interocular suppression should involve extrastriate regions far removed from striate cortex,^55^ although it has been suggested that it might involve a deficit to selective attention,^35^ implicating prefrontal^56^^,^^57^ and/or posterior parietal areas.^56^^,^^57^ For attention to play a role in amblyopic suppression, it would need to be established that the attentive feedback from higher cortical area has access to monocular signals. A recent study in binocular normal individuals using a binocular rivalry paradigm provides support for this assertion.^58^ Our results suggest a role for the frontal region in interocular suppression that is not related to acuity, suggesting a nonspecific, higher level modulation. Further studies are necessary to better identify regions involved in interocular suppression, their links with higher level processing such as attention, and their functional relationship with the occipital cortex.
